# Ki-67 Proliferation Index in Pulmonary Neuroendocrine Neoplasms: Interobserver Agreement Among Pathologists and Comparison of Two Artificial Intelligence-Based Image Analysis Systems

**DOI:** 10.3390/biomedicines14030627

**Published:** 2026-03-11

**Authors:** Gizem Teoman, Zeynep Turkmen Usta, Zeynep Sagnak Yilmaz, Safak Ersoz

**Affiliations:** Department of Medical Pathology, Faculty of Medicine, Karadeniz Technical University, 61080 Trabzon, Turkey; zeynepturkmen@ktu.edu.tr (Z.T.U.); zeynep.sagnak@ktu.edu.tr (Z.S.Y.); sersoz@ktu.edu.tr (S.E.)

**Keywords:** lung, neuroendocrine neoplasms, Ki-67, digital pathology, artificial intelligence

## Abstract

**Background/Objectives:** Although Ki-67 is not formally incorporated into the grading system of pulmonary neuroendocrine neoplasms (PNENs), it is widely used as an adjunct marker to reflect proliferative activity and support diagnostic stratification. Manual Ki-67 assessment is subject to interobserver variability and methodological limitations. This study aimed to evaluate the reliability and performance of two artificial intelligence (AI)-based image analysis systems in Ki-67 index assessment and to compare their results with expert pathologist evaluation in pulmonary neuroendocrine tumors. **Methods:** A total of 63 pulmonary neuroendocrine neoplasm cases, including typical carcinoid (n = 29), atypical carcinoid (n = 13), and large cell neuroendocrine carcinoma (n = 21), were retrospectively analyzed. Ki-67 proliferation indices were independently assessed by four pathologists within predefined hotspot regions, counting approximately 2000 tumor cells per case. The same regions were analyzed using two AI-based image analysis systems (Roche uPath Ki-67 and Virasoft Virasight Ki-67). Interobserver agreement among pathologists was evaluated using the intraclass correlation coefficient (ICC), and concordance between manual and AI-based assessments was assessed using Spearman’s correlation and linear regression analyses. To account for potential scanner/platform effects, slides were digitized using two different whole-slide scanners (VENTANA DP^®^ 600 and Leica Aperio AT2), and color normalization and quality control procedures were applied prior to AI-based analysis. For clinical interpretability, Ki-67 indices were stratified into categorical groups based on tumor subtype-specific thresholds (0–<10%: low, 10–25%: intermediate, >25%: high), and agreement between manual and AI-based categorical scoring was evaluated using Cohen’s kappa coefficient. **Results:** Among the 63 pulmonary neuroendocrine neoplasm cases, Ki-67 proliferation indices varied across tumor subtypes, with typical carcinoids showing low, atypical carcinoids intermediate, and large cell neuroendocrine carcinomas high proliferative activity. Interobserver agreement among four pathologists was excellent (ICC = 0.998, 95% CI: 0.996–0.998). Strong correlations were observed between manual Ki-67 assessments and AI-derived indices, with Spearman correlation coefficients of 0.961 (95% CI: 0.918–0.982) for Roche AI and 0.904 (95% CI: 0.821–0.949) for Virasoft AI, and 0.926 (95% CI: 0.842–0.968) between the two AI systems. Bland–Altman analyses demonstrated minimal mean differences and most cases within the 95% limits of agreement, indicating high concordance without systematic bias. Categorical agreement analysis, using subtype-specific Ki-67 thresholds (0–<10%: low; 10–25%: intermediate; >25%: high), showed excellent concordance between manual and AI-based scoring (Cohen’s kappa 0.877 for Roche AI and 0.827 for Virasoft AI; *p* < 0.001), confirming the clinical interpretability and reproducibility of AI-based Ki-67 assessment. **Conclusions:** AI-based Ki-67 index assessment shows strong concordance with expert pathologist evaluation and reflects biologically relevant differences among pulmonary neuroendocrine neoplasm subtypes. These results suggest that AI-assisted Ki-67 analysis may serve as a reproducible and objective adjunct to routine diagnostic practice in pulmonary neuroendocrine tumors.

## 1. Introduction

Pulmonary neuroendocrine neoplasms (NENs) comprise a biologically and clinically heterogeneous group of neoplasms that arise from neuroendocrine cells of the respiratory epithelium. According to the current World Health Organization (WHO) classification, pulmonary NENs are categorized into four major subtypes based on morphological features, mitotic activity, and the presence of necrosis: typical carcinoid (TC), atypical carcinoid (AC), large cell neuroendocrine carcinoma (LCNEC), and small cell lung carcinoma (SCLC) [1]. These entities exhibit markedly different clinical behaviors, ranging from indolent tumors with favorable prognosis to highly aggressive carcinomas associated with poor survival outcomes. Therefore, accurate classification and reliable prognostic stratification are critical for guiding therapeutic decision-making and predicting patient outcomes [2].

The Ki-67 proliferation index, which reflects the proportion of tumor cells actively engaged in the cell cycle, is a widely accepted biomarker of cellular proliferation and tumor aggressiveness. In neuroendocrine neoplasms, Ki-67 has long been established as a key parameter for grading and prognostic evaluation, particularly in gastroenteropancreatic NENs. Although pulmonary NENs are primarily classified based on histomorphological criteria, accumulating evidence suggests that Ki-67 provides valuable adjunctive information for distinguishing between low-grade and high-grade tumors, identifying biologically aggressive lesions, and supporting prognostic assessment, especially in diagnostically challenging cases [3]. Consequently, Ki-67 immunohistochemistry is increasingly incorporated into routine diagnostic practice for pulmonary NENs.

Despite its clinical utility, manual assessment of Ki-67 immunohistochemical staining remains a subjective and labor-intensive process. Significant interobserver variability has been reported among pathologists, mainly attributable to differences in hot spot selection, variability in defining tumor versus non-tumor cells, and discrepancies in manual counting methods. These challenges are particularly pronounced in tumors with heterogeneous staining patterns or borderline proliferation indices, where small differences in counting may lead to clinically meaningful changes in interpretation. Such variability may compromise reproducibility, limit standardization across institutions, and ultimately impact diagnostic consistency and patient management [3].

In recent years, advances in digital pathology and artificial intelligence (AI)-based image analysis have introduced new opportunities for objective and reproducible quantification of immunohistochemical biomarkers. AI-driven algorithms, particularly those based on machine learning and deep learning techniques, enable automated detection of tumor nuclei, identification of Ki-67-positive cells, and precise calculation of proliferation indices within selected regions of interest. These systems offer the potential to reduce observer-dependent bias, improve analytical consistency, and enhance workflow efficiency in routine pathology practice. Previous studies have demonstrated high concordance between AI-assisted and manual Ki-67 assessments in various tumor types, most notably in gastrointestinal and pancreatic neuroendocrine neoplasms [4].

However, data regarding the performance and reliability of AI-based Ki-67 analysis in pulmonary NENs remain limited. Furthermore, comparative evaluations of different AI image analysis systems and their agreement with pathologist-derived assessments are scarce. Given the heterogeneity of pulmonary NENs and the critical role of Ki-67 in their evaluation, systematic assessment of interobserver agreement among pathologists and validation of AI-assisted approaches are of particular importance [5].

In this context, the present study aimed to evaluate interobserver agreement in manual Ki-67 assessment among pathologists and to compare these results with Ki-67 proliferation indices obtained using two different artificial intelligence-based image analysis systems in pulmonary neuroendocrine tumors. By analyzing concordance, reliability, and reproducibility across manual and AI-assisted methods, we sought to determine the potential role of AI as a supportive tool in routine diagnostic practice and to assess whether AI-based systems can contribute to more standardized and objective Ki-67 evaluation in pulmonary NENs.

## 2. Materials and Methods

### 2.1. Ethical Approval

This retrospective study was conducted in accordance with the ethical principles of the Declaration of Helsinki and was approved by the Ethics Committee of Karadeniz Technical University Faculty of Medicine (Approval number: 24237859-178). Authorization for the use of archived pathology materials was obtained from the Hospital Directorate. Owing to the retrospective design, informed consent was waived. All patient data were anonymized prior to analysis, and no identifiable information was included in the study.

### 2.2. Case Selection

A total of 63 pulmonary neuroendocrine tumor cases diagnosed between 2020 and 2025 at the Department of Medical Pathology, Karadeniz Technical University Faculty of Medicine, were retrospectively included. The study cohort consisted of 29 typical carcinoids (TCs), 13 atypical carcinoids (ACs), and 21 large cell neuroendocrine carcinomas (LCNECs), classified according to the current World Health Organization criteria.

Only cases with available formalin-fixed, paraffin-embedded tissue blocks obtained from surgical resections or excisional biopsies were eligible for inclusion. Small biopsy specimens and consultation cases were excluded to ensure sufficient tissue for reliable Ki-67 hot-spot evaluation. Small cell lung carcinoma cases were not included, as these tumors were diagnosed exclusively on small needle biopsy specimens without subsequent surgical resection.

Given the relative rarity of pulmonary neuroendocrine tumors, particularly atypical carcinoids, this study was designed as an exploratory analysis. Therefore, a formal sample size calculation was not performed.

### 2.3. Manual Ki-67 Assessment

Four experienced pathologists independently evaluated the Ki-67 proliferation index within the predefined hot-spot region. All pathologists were blinded to each other’s results, to the AI-derived measurements, and to all clinical and pathological data.

Manual Ki-67 assessment was performed by counting approximately 2000 tumor cells within the annotated hot-spot area [4]. The Ki-67 proliferation index was calculated as the percentage of tumor cell nuclei showing positive immunoreactivity. This predefined hotspot approach was purposefully adopted to standardize the evaluation across observers, minimize variability related to hotspot selection, and ensure consistency when comparing manual and AI-based scoring. This approach means that the measured interobserver agreement reflects concordance in counting/classification within the same ROI, rather than agreement in hotspot selection, which may vary in routine practice.

### 2.4. Digital Slide Scanning and AI-Based Image Analysis

For AI-based analysis, Ki-67-stained slides were digitized using two different whole-slide scanning platforms, according to the requirements of each algorithm.

For the Roche algorithm, slides were scanned using the VENTANA DP^®^ 600 whole-slide scanner (Roche Diagnostics, Mannheim, Germany). Digital images were analyzed using the uPath Ki-67 image analysis algorithm (version 1.0.0.5; Roche Diagnostics), which is based on a convolutional neural network (CNN) architecture for automated nuclear detection, segmentation, and classification. Within the predefined hot-spot region, the algorithm automatically classified tumor cell nuclei as Ki-67-positive or negative using predefined optical density and chromogen intensity thresholds. The Ki-67 labeling index was calculated based on the evaluation of approximately 2000 tumor cells.

For the Turkish-developed algorithm, slides were scanned using the Leica Aperio AT2 whole-slide scanner (Leica Biosystems, Wetzlar, Germany) and uploaded to the digital analysis platform. Ki-67 quantification was performed using the Virasoft Virasight Ki-67 algorithm, an artificial intelligence-based image analysis system utilizing machine learning-assisted nuclear segmentation and classification techniques. Analysis was restricted to the same predefined hot-spot region used for manual assessment and Roche algorithm analysis. The Ki-67 proliferation index was calculated as the percentage of positively stained tumor nuclei among approximately 2000 evaluated tumor cells.

Although Ki-67-stained slides were digitized using two different whole-slide scanners (VENTANA DP^®^ 600 for the Roche algorithm and Leica Aperio AT2 for the Virasoft algorithm), both systems provide high-resolution imaging with comparable optical quality. To minimize potential platform-related variability, color normalization and quality control procedures were applied prior to AI-based analysis. These steps ensured that differences in scanner hardware did not substantially affect the performance or reliability of Ki-67 quantification across the two platforms.

All immunohistochemical staining procedures were performed in the same laboratory using identical protocols. Prior to AI-based analysis, digital slides were reviewed for image quality, focus, staining consistency, and background artifacts to minimize technical variability.

### 2.5. Statistical Analysis

Statistical analyses were performed using IBM SPSS Statistics software (version 27.0; IBM Corp., Armonk, NY, USA). Interobserver agreement among pathologists was assessed using the intraclass correlation coefficient (ICC). Intraclass correlation coefficients (ICCs) were calculated using a two-way mixed-effects model, in which subject effects were treated as random and measurement effects as fixed, with absolute agreement. Ninety-five percent confidence intervals (CIs) were also reported. Similarly, 95% CIs were provided for correlation analyses, obtained using bootstrap resampling where appropriate. Concordance between manual Ki-67 assessments and AI-derived results, as well as agreement between the two AI algorithms, was evaluated using Spearman’s rank correlation coefficient. To assess the potential effect of scanner/platform differences on algorithm performance and generalizability, results were compared across the two AI systems and with manual scoring by pathologists. Bland–Altman and correlation analyses were used to evaluate agreement, while color normalization and quality control procedures were applied prior to AI-based analysis to minimize platform-related variability.

To evaluate the clinical interpretability of Ki-67 scoring, cases were stratified into categorical groups based on tumor subtype-specific thresholds informed by previous literature [6]: 0–<10% (low), 10–25% (intermediate), and >25% (high). Categorical agreement between manual and AI-based Ki-67 scoring (Roche and Virasoft) was assessed using Cohen’s kappa coefficient, with statistical significance set at *p* < 0.05.

No artificial intelligence-assisted tools, including large language models, chatbots, or image generation software, were used in the preparation of this manuscript.

## 3. Results

### 3.1. Case Distribution

A total of 63 pulmonary neuroendocrine neoplasm cases were included in the study. Of these, 29 cases (46.0%) were classified as typical carcinoid (TC), 13 cases (20.6%) as atypical carcinoid (AC), and 21 cases (33.3%) as large cell neuroendocrine carcinoma (LCNEC).

### 3.2. Manual Ki-67 Assessment by Pathologists

Ki-67 proliferation indices obtained from manual assessment by four pathologists demonstrated distinct distributions across tumor subtypes. For typical carcinoid tumors, the median Ki-67 index was 2.09%, with values ranging from a minimum of 0.55% to a maximum of 7.75%. The standard deviation for TC cases was 1.63, reflecting a relatively narrow dispersion of values. In atypical carcinoid tumors, the median Ki-67 index was 16.2%, with a minimum value of 11% and a maximum value of 21.5%. The standard deviation in this subgroup was 1.63. Large cell neuroendocrine carcinomas exhibited substantially higher Ki-67 indices, with a median value of 63.7%, ranging from 35.2% to 85%. The standard deviation for LCNEC cases was 14.43, indicating greater variability in proliferative activity within this high-grade subgroup.

### 3.3. AI Quantification

For Ki-67 index assessment, hotspot areas were initially selected by an experienced pathologist and subsequently analyzed using two different artificial intelligence-based digital image analysis systems: Roche AI and Virasoft AI. In a representative case, the selected region of interest (ROI) contained approximately 2000 tumor cell nuclei.

**Figure 1:**
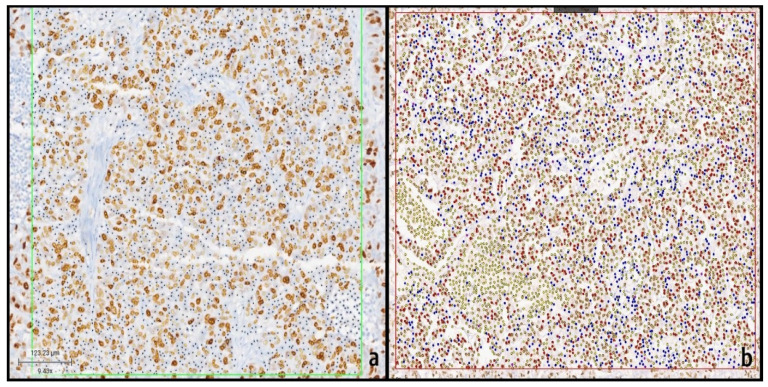
Representative regions of interest (ROI) (inside of green [Roche algorithm] and red boxes [Virasoft algorithm]) demonstrating Ki-67 index assessment using two image analysis algorithms. (**a**) In the Roche algorithm, Ki-67-positive tumor nuclei are marked with yellow dots and negative nuclei with black dots, and the labeling index is calculated using a binary classification approach. (**b**) In the Virasoft algorithm, nuclei are similarly marked with colored dots according to staining intensity: strongly positive (red), moderately positive (orange), weakly positive (yellow), and negative (blue). The Ki-67 index is calculated as the proportion of positively stained nuclei among all detected nuclei within the same ROI.

Using the Roche AI algorithm, Ki-67-positive nuclei showing brown nuclear staining were automatically detected and marked with yellow dots, whereas Ki-67-negative nuclei were identified and marked with black dots. Based on this binary classification, the Ki-67 labeling index was calculated as the proportion of positively stained nuclei among all detected nuclei, yielding a Ki-67 index of 45.7% in the analyzed ROI (Figure 1a).

In contrast, analysis with the Virasoft AI system applied a more detailed classification of Ki-67-positive nuclei according to staining intensity. Positively stained nuclei were subdivided into strongly, moderately, and weakly stained categories and were marked in red, orange, and yellow, respectively, while Ki-67-negative nuclei were marked in blue. The Virasoft AI algorithm automatically calculated the Ki-67 index as the ratio of all positively stained nuclei (regardless of staining intensity) to the total number of detected nuclei. Using this approach, the Ki-67 index for the same ROI was calculated as 48.2% (Figure 1b).

### 3.4. Interobserver Agreement Among Pathologists

Interobserver agreement among the four pathologists was assessed using the intraclass correlation coefficient (ICC). Overall agreement across all 63 cases was excellent, with an ICC value of 0.998 (95% CI: 0.996–0.998), indicating near-perfect concordance.

When analyzed by tumor subtype, interobserver agreement remained high. The ICC was 0.958 (95% CI: 0.915–0.977) for typical carcinoid tumors, 0.840 (95% CI: 0.760–0.902) for atypical carcinoid tumors, and 0.977 (95% CI: 0.956–0.988) for large cell neuroendocrine carcinomas. Although slightly lower agreement was observed in the atypical carcinoid subgroup, concordance remained within the range considered good to excellent.

### 3.5. Comparison Between Manual Assessment and AI-Based Analysis

Agreement between manual Ki-67 assessment by pathologists and AI-based image analysis was evaluated using Spearman’s rank correlation coefficient.

A strong positive correlation was observed between pathologist-derived Ki-67 indices and results obtained using the Roche AI algorithm, with a correlation coefficient of 0.961 (95% CI: 0.918–0.982), indicating excellent concordance. Similarly, Ki-67 indices generated by the Virasoft Virasight Ki-67 algorithm demonstrated a strong correlation with manual assessments by pathologists, with a correlation coefficient of 0.904 (95% CI: 0.821–0.949). Furthermore, comparison between the two AI-based image analysis systems revealed a high level of agreement, with a Spearman correlation coefficient of 0.926 (95% CI: 0.842–0.968).

**Figure 2:**
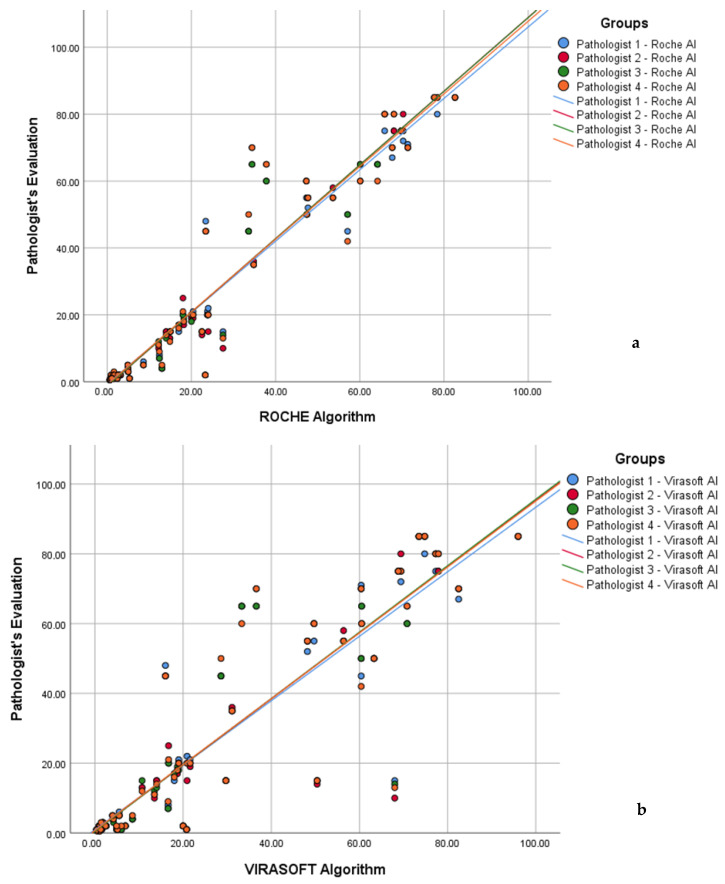
Scatter plots demonstrating the correlation between Ki-67 index values assessed by four pathologists and those calculated by (**a**) the Roche algorithm and (**b**) the Virasoft algorithm. Each point represents an individual case, with different colors corresponding to individual pathologists. Solid lines indicate linear regression fits. High coefficients of determination (R^2^ = 0.921–0.940 for Roche; R^2^ = 0.813–0.825 for Virasoft) indicate strong agreement between pathologist-based and algorithm-derived Ki-67 measurements.

Scatter plot analysis demonstrated strong linear correlations between Ki-67 index values assessed by four pathologists and those calculated using both Roche AI and Virasoft AI systems across the full range of proliferation indices. Linear regression analysis revealed higher agreement with Roche AI (R^2^ = 0.921–0.940) compared with Virasoft AI (R^2^ = 0.813–0.825), indicating consistently strong concordance between manual hotspot-based assessment and automated AI-derived measurements. Overall, these findings support the reliability and reproducibility of AI-based Ki-67 index calculation, with Roche AI showing slightly superior concordance with expert pathologist evaluation (Figure 2a,b).

**Figure 3:**
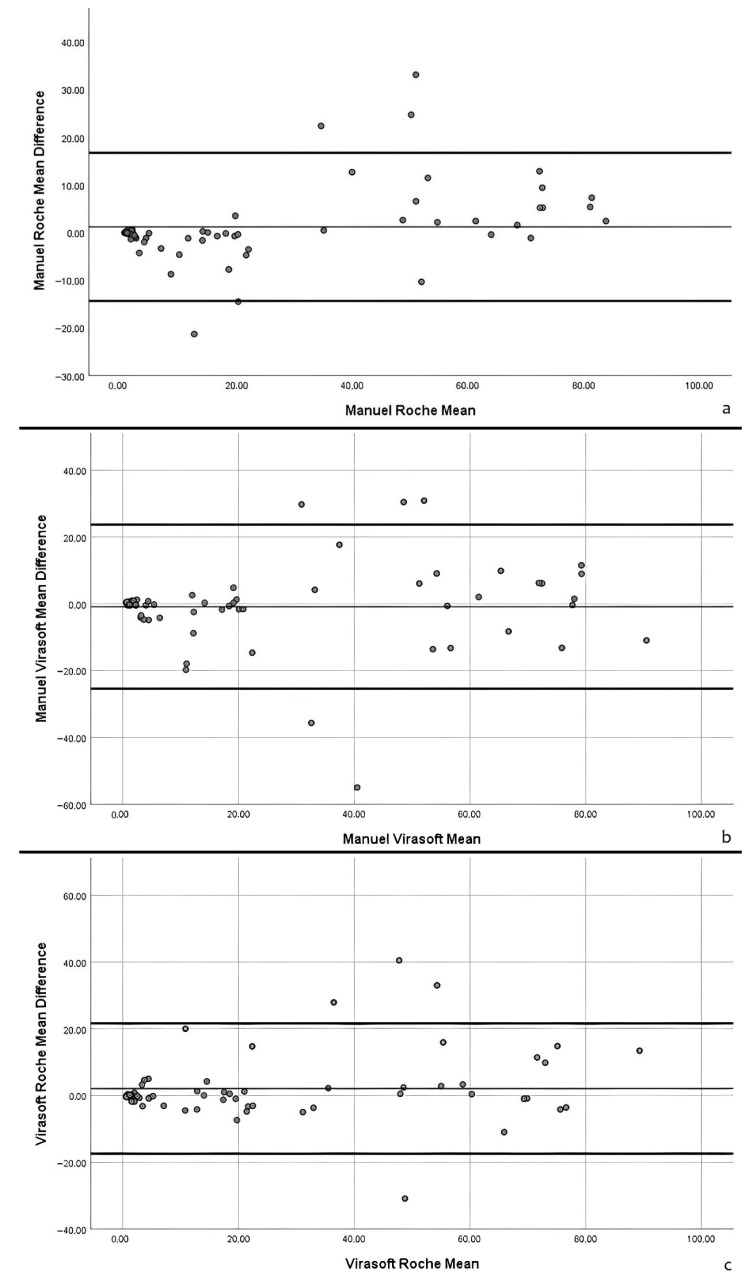
Bland–Altman plots comparing Ki-67 scoring methods. (**a**–**c**) Bland–Altman analyses illustrating the agreement between manual and AI-based Ki-67 scoring. (**a**) manual vs. Roche; (**b**) manual vs. Virasoft; (**c**) Roche vs. Virasoft. The solid line represents the mean difference between the two methods, while the other two lines indicate the 95% limits of agreement. Most cases fall within these limits.

Agreement between manual and AI-based Ki-67 scoring was further evaluated using Bland–Altman plots for the key comparisons: manual vs. Roche, manual vs. Virasoft, and Roche vs. Virasoft (Figure 3a–c). The plots illustrate the mean difference and 95% limits of agreement for each comparison.

For manual vs. Roche, the mean difference was small, and the majority of cases fell within the 95% limits of agreement, indicating good concordance between manual counting and Roche AI measurements. Similarly, manual vs. Virasoft showed that most cases were within the agreement limits, although a few outliers were observed at higher Ki-67 values. Finally, the Roche vs. Virasoft comparison demonstrated that the two AI systems were largely consistent, with the majority of cases falling within the acceptable limits.

Overall, these analyses indicate that both AI systems provide Ki-67 scores that are largely concordant with manual counting, and there was no substantial systematic over- or underestimation observed at low or high Ki-67 values. These results support the reliability of AI-based scoring for standardized Ki-67 assessment in pulmonary neuroendocrine neoplasms.

Box plot analyses demonstrated a clear and consistent stratification of Ki-67 index values across diagnostic subgroups in both pathologist-based assessment and AI-assisted analyses (Roche AI and Virasoft AI). Across all three evaluation methods, Ki-67 indices exhibited a stepwise increase from typical carcinoid (TC) to large cell neuroendocrine carcinoma (LCNEC), reflecting progressively increasing proliferative activity.

TC cases showed uniformly low Ki-67 values with minimal variability, whereas atypical carcinoid (AC) cases demonstrated intermediate Ki-67 indices with relatively narrow distributions. In contrast, LCNEC cases displayed markedly elevated Ki-67 indices accompanied by broader interquartile ranges, indicating both high proliferative activity and greater intragroup heterogeneity.

**Figure 4:**
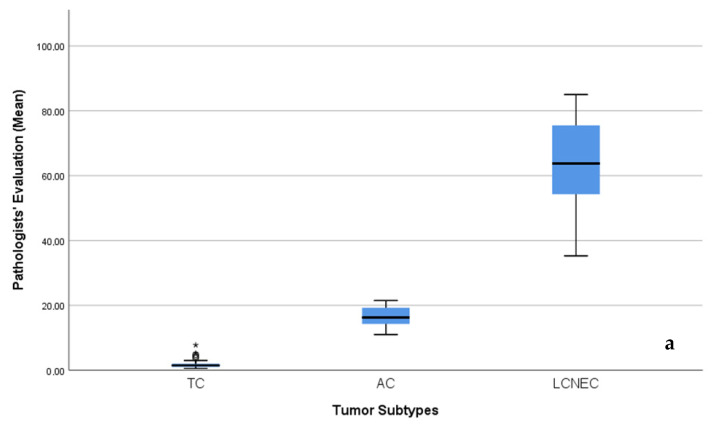

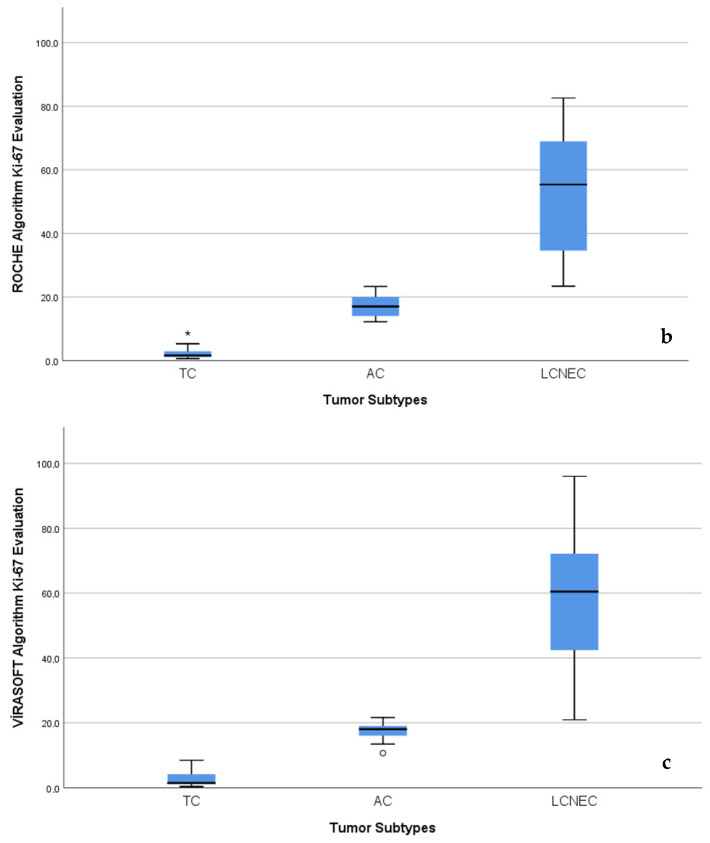
Box plots showing the distribution of Ki-67 index values across pulmonary neuroendocrine neoplasm subtypes—typical carcinoid (TC), atypical carcinoid (AC), and large cell neuroendocrine carcinoma (LCNEC)—based on (**a**) pathologist evaluation, (**b**) Roche algorithm analysis, and (**c**) Virasoft algorithm analysis. In all panels, the central line represents the median, the boxes indicate the interquartile range (IQR), and the * denote the minimum and maximum values; outliers (in panel **b**) are shown as individual points.

The distinct separation among tumor subtypes observed consistently across all assessment methods underscores the capability of both AI algorithms to reliably discriminate between TC and LCNEC based on the Ki-67 proliferation index, showing strong concordance with pathologist-based evaluation (Figure 4a–c).

### 3.6. Categorical Agreement Analysis

To translate Ki-67 scores into clinically meaningful categories, cases were stratified based on tumor subtype-specific thresholds derived from previous literature [6]: 0–<10% (low), 10–25% (intermediate), and >25% (high). Categorical agreement between manual Ki-67 assessment and Roche AI scoring was excellent, with a Cohen’s kappa of 0.877 (*p* < 0.001), while agreement between manual assessment and Virasoft AI was similarly high (kappa = 0.827, *p* < 0.001). These findings demonstrate that both AI-based systems reliably reproduce clinically relevant Ki-67 categories and support their potential utility for standardized diagnostic and prognostic evaluation of pulmonary neuroendocrine neoplasms. The categorical stratification aligned closely with the continuous Ki-67 distributions observed across tumor subtypes, with typical carcinoid tumors predominantly classified as low, atypical carcinoids as intermediate, and large cell neuroendocrine carcinomas as high, consistent with both pathologist- and AI-based analyses.

## 4. Discussion

Accurate assessment of the Ki-67 proliferation index is increasingly recognized as a valuable adjunct in the evaluation of pulmonary neuroendocrine neoplasms (PNENs), particularly for supporting diagnostic stratification and reflecting biological proliferative activity, although it is not currently included as a formal grading criterion in lung neuroendocrine neoplasms [7]. However, manual Ki-67 evaluation is inherently subject to interobserver variability, differences in hotspot selection, and time-consuming counting procedures, particularly in tumors with heterogeneous proliferative activity [8]. In this study, we performed Ki-67 scoring within predefined hotspot regions, which allowed standardization across pathologists and minimized variability related to hotspot selection. In this context, artificial intelligence-based image analysis systems have emerged as promising tools to improve objectivity, reproducibility, and efficiency in Ki-67 assessment. It should be noted that the interobserver agreement reported in this study primarily reflects concordance in counting/classification within the same hotspot ROI, rather than agreement in hotspot selection, which may vary in routine clinical practice.

In the present study, we demonstrated excellent interobserver agreement among four experienced pathologists, with an overall ICC of 0.998 (95% CI: 0.996–0.998), confirming that standardized hotspot selection and counting of a fixed number of tumor cells can yield highly reproducible results even in challenging tumor subtypes. When analyzed by tumor subtype, interobserver agreement remained high, with ICC values of 0.958 (95% CI: 0.915–0.977) for typical carcinoid tumors, 0.840 (95% CI: 0.760–0.902) for atypical carcinoid tumors, and 0.977 (95% CI: 0.956–0.988) for large cell neuroendocrine carcinomas. Nevertheless, slightly reduced agreement in atypical carcinoid tumors underscores the well-recognized diagnostic gray zone between low- and intermediate-grade pulmonary neuroendocrine neoplasms, where subtle differences in proliferative activity may significantly influence classification and clinical management.

Both AI-based systems evaluated in this study—Roche uPath Ki-67 and Virasoft Virasight Ki-67—showed strong concordance with manual pathologist assessment. Correlation analyses demonstrated excellent agreement between Roche AI and pathologist-derived Ki-67 indices (Spearman r = 0.961, 95% CI: 0.918–0.982), and a similarly strong correlation for Virasoft AI (r = 0.904, 95% CI: 0.821–0.949). Comparison between the two AI platforms revealed high concordance (r = 0.926, 95% CI: 0.842–0.968). The high level of agreement observed between the two AI platforms themselves further supports the robustness of automated Ki-67 quantification when standardized hotspot regions are used. Notably, Roche AI showed slightly higher coefficients of determination in linear regression analysis compared with Virasoft AI, which may be attributable to differences in algorithm architecture, nuclear segmentation strategies, and handling of staining intensity.

Although Ki-67-stained slides were digitized using two different whole-slide scanners (VENTANA DP^®^ 600 for the Roche algorithm and Leica Aperio AT2 for the Virasoft algorithm), both systems provide high-resolution imaging with comparable optical quality. Color normalization and quality control procedures were applied prior to analysis to minimize potential platform-related variability. Bland–Altman analyses demonstrated that the minor differences between scanners did not substantially affect the reliability of Ki-67 quantification. Taken together, these findings indicate that AI-based Ki-67 algorithms produce reliable and reproducible results across different scanning platforms, supporting their generalizability.

Importantly, both AI systems effectively reproduced the biologically expected stratification of Ki-67 indices across tumor subtypes. Typical carcinoid tumors consistently exhibited low Ki-67 values with minimal variability, atypical carcinoids demonstrated intermediate proliferation indices, and large cell neuroendocrine carcinomas showed markedly elevated and heterogeneous Ki-67 values. This clear stepwise increase in proliferative activity across tumor grades was observed not only in pathologist-based assessment but also in both AI-derived analyses, highlighting the ability of AI algorithms to reliably discriminate low- and high-grade pulmonary neuroendocrine neoplasms. These findings align with previous studies emphasizing the diagnostic and prognostic relevance of Ki-67 in pulmonary neuroendocrine neoplasms, particularly in distinguishing carcinoid tumors from high-grade neuroendocrine carcinomas [9].

Beyond diagnostic reproducibility, AI-assisted Ki-67 evaluation offers several practical advantages. Automated analysis reduces observer-dependent variability, enables rapid assessment of large tumor areas, and provides standardized quantitative outputs that may facilitate multi-center studies and integration into digital pathology workflows [10]. Such advantages are particularly relevant given the increasing interest in incorporating Ki-67 into refined grading systems and treatment algorithms for pulmonary neuroendocrine neoplasms [11].

This study has several limitations. Its retrospective design and relatively limited sample size—especially for atypical carcinoid tumors—reflect the rarity of pulmonary neuroendocrine neoplasms and may limit the generalizability of the findings. Small cell lung carcinoma cases were excluded due to the lack of surgical specimens, precluding evaluation of AI performance in this subgroup. Additionally, clinical outcome data were not included, preventing assessment of the prognostic impact of AI-derived Ki-67 indices. Furthermore, while manual Ki-67 counting is available in virtually all pathology laboratories and continues to provide reproducible results with optimal interobserver concordance, AI-based image analysis systems are more complex and not yet widely accessible, which may limit their immediate implementation in routine practice. Future prospective studies with larger cohorts and survival analyses are warranted to further validate the clinical utility of AI-based Ki-67 quantification.

## 5. Conclusions

In conclusion, our findings demonstrate that both Roche AI and Virasoft AI provide reliable, reproducible, and biologically meaningful Ki-67 index measurements that closely mirror expert pathologist assessment. AI-assisted Ki-67 analysis represents a valuable adjunct to routine diagnostic practice and holds significant potential for standardizing proliferation assessment in pulmonary neuroendocrine neoplasms.

## Data Availability

The data presented in this study are not publicly available due to ethical and privacy restrictions. The data may be made available from the corresponding author upon reasonable request and with permission of the Scientific Research Ethics Committee of Karadeniz Technical University Faculty of Medicine.
